# Activated Phosphoinositide 3-Kinase Delta Syndrome 1: Clinical and Immunological Data from an Italian Cohort of Patients

**DOI:** 10.3390/jcm9103335

**Published:** 2020-10-17

**Authors:** Giulio Tessarin, Stefano Rossi, Manuela Baronio, Luisa Gazzurelli, Michael Colpani, Alessio Benvenuto, Fiammetta Zunica, Fabio Cardinale, Baldassarre Martire, Letizia Brescia, Giorgio Costagliola, Laura Luti, Gabriella Casazza, Maria Cristina Menconi, Francesco Saettini, Laura Palumbo, Maria Federica Girelli, Raffaele Badolato, Gaetana Lanzi, Marco Chiarini, Daniele Moratto, Antonella Meini, Silvia Giliani, Maria Pia Bondioni, Alessandro Plebani, Vassilios Lougaris

**Affiliations:** 1 Pediatrics Clinic and “A. Nocivelli” Institute for Molecular Medicine, Department of Clinical and Experimental Sciences, University of Brescia, ASST Spedali Civili of Brescia, 25123 Brescia, Italy; giulio.tessarin@gmail.com (G.T.); stefreds90@gmail.com (S.R.); manuelabaronio@hotmail.it (M.B.); l.gazzurelli@gmail.com (L.G.); m.colpani002@unibs.it (M.C.); Alessio.benvenuto.lssa@gmail.com (A.B.); fiammetta.zunica@gmail.com (F.Z.); raffaele.badolato@unibs.it (R.B.); alessandro.plebani@unibs.it (A.P.); 2 Allergy, Immunology and Pediatric Pulmonology Unit, “Policlinico-Giovanni XXII” Hospital, University of Bari, 70126 Bari, Italy; fabiocardinale@libero.it; 3 Pediatrics Unit, Monsignor Dimiccoli Hospital, 70051 Barletta, Italy; baldo.martire@gmail.com; 4 Pediatric Oncohematology Unit, Ospedale Santissima Annunziata, 74121 Taranto, Italy; letiziapomponia.brescia@asl.taranto.it; 5 Division of Pediatrics, University of Pisa, 56126 Pisa, Italy; giorgio.costagliola@hotmail.com; 6 Pediatric Oncology and Hematology, University of Pisa, 56126 Pisa, Italy; laura.luti@tin.it (L.L.); g.casazza@ao-pisa.toscana.it (G.C.); mc.menconi@gmail.com (M.C.M.); 7 Pediatric Hematology Oncology Unit, Department of Pediatrics, University of Milano Bicocca, MBBM Foundation, 20900 Monza, Italy; francescosaettini@yahoo.it; 8 Pediatrics Clinic, ASST-Spedali Civili of Brescia, 25123 Brescia, Italy; palumbolauretta@gmail.com (L.P.); federicagirelli@hotmail.it (M.F.G.); antonella.meini@yahoo.it (A.M.); 9 “Angelo Nocivelli” Institute for Molecular Medicine, ASST Spedali Civili, Department of Molecular and Translational Medicine, University of Brescia, 25123 Brescia, Italy; g_lanzi@hotmail.com; 10 Clinical Chemical Analysis Central Laboratory, ASST Spedali Civili of Brescia, 25123 Brescia, Italy; marco.chiarini@asst-spedalicivili.it (M.C.); daniele.moratto@gmail.com (D.M.); 11 Cytogenetic and Medical Genetics Unit and “A. Nocivelli” Institute for Molecular Medicine, Department of Molecular and Translational Medicine, University of Brescia, ASST Spedali Civili of Brescia, 25123 Brescia, Italy; silvia.giliani@unibs.it; 12 Pediatric Radiology, University of Brescia, ASST Spedali Civili di Brescia, 25123 Brescia, Italy; mariapiabondioni@gmail.com

**Keywords:** activated phosphoinositide 3-kinase delta syndrome 1, lymphoproliferation, primary combined immune deficiency, p110δ, *PIK3CD*, PI3K

## Abstract

Activated phosphoinositide 3-kinase delta syndrome 1 (APDS-1) is a recently described inborn error of immunity caused by monoallelic gain-of-function mutations in the *PIK3CD* gene. We reviewed for the first time medical records and laboratory data of eight Italian APDS-1 patients. Recurrent sinopulmonary infections were the most common clinical feature at onset of disease. Seven patients presented lymphoproliferative disease, at onset or during follow-up, one of which resembled hemophagocytic lymphohistiocytosis (HLH). Genetic analysis of the *PIK3CD* gene revealed three novel mutations: functional testing confirmed their activating nature. In the remaining patients, the previously reported variants *p*.E1021K (*n* = 4) and *p*.E525A (*n* = 1) were identified. Six patients were started on immunoglobulin replacement treatment (IgRT). One patient successfully underwent hematopoietic stem cell transplantation (HSCT), with good chimerism and no GVHD at 21 months post-HSCT. APDS-1 is a combined immune deficiency with a wide variety of clinical manifestations and a complex immunological presentation. Besides IgRT, specific therapies targeting the PI3Kδ pathway will most likely become a valid aid for the amelioration of patients’ clinical management and their quality of life.

## 1. Introduction

Activated Phosphoinositide 3-Kinase Delta Syndrome-1 (APDS-1, Online Mendelian Inheritance in Man (OMIM #615513) is a recently described inborn error of immunity (IEI) [1,2]. The disease is caused by gain-of-function (GOF) mutations in the *PIK3CD* gene located at chromosome 1p36 (OMIM * 602839), coding for the phosphoinositide 3-kinase (PI3K) catalytic subunit p110δ [3].

PI3Ks have been divided into three classes according to their structural characteristics and substrate specificity [4]. Of these, the most commonly studied are the class I enzymes that are activated directly by cell surface receptors [5]. Class I PI3Ks are mainly expressed in leukocytes and play a major role in diverse cell functions including cell growth, proliferation, differentiation and survival [6]. They are formed by heterodimers comprising a regulatory (p85α, p55α, p50α, p85β, or p55γ) and a catalytic subunit (p110α, β, or δ) [6]. GOF mutations on the p110δ subunit lead to a downstream hyperactivation of the AKT/mammalian target of rapamycin (mTOR) signaling pathway [7].

Clinical hallmarks of the disease are recurrent sinopulmonary infections, severe or persistent viral infections, autoimmunity (e.g., cytopenia, arthritis, enteropathy) and chronic benign lymphoproliferation with increased risk of developing lymphomas [8,9,10,11]. APDS-1 patients display defects in both B- and T-cells: usual findings include lymphopenia, normal to elevated serum immunoglobulin M (IgM) levels, and reduced levels of immunoglobulin G (IgG) with impaired ability to produce antibodies against vaccines [12,13,14,15]. A characteristic feature of APDS-1 patients is T cell immunosenescence with raised CD8^+^CD57^+^ cells and CD8^+^ T-cells skewed towards effector memory [16]. Genetic analysis of APDS-1 patients revealed in the vast majority of cases the recurrent E1021K monoallelic mutation, with a limited number of novel variants reported since the identification of the disorder [17].

Symptomatic therapeutic strategies for APDS-1 recall the ones used for Common Variable Immune Deficiency (CVID): immunoglobulin replacement treatment (IgRT) and antibiotic cycles to prevent and treat infections, immunosuppression to control lymphoproliferation and autoimmune manifestations [9,18], while the hyper-activation of PI3K signaling pathway in APDS-1 enabled targeted therapy in the form of mTOR inhibitors [19]. Recently, a clinical trial using the p110δ selective inhibitor leniolisib has been undertaken: leniolisib greatly reduced lymphoproliferation and autoimmune phenomena, with no reports of significant adverse events [20]. Hematopoietic stem cell transplantation (HSCT) has been used for a limited number of affected patients and available data suggest that in a limited number of cases HSCT may become a valid option based on clinical conditions and medical treatment refractory complications [21,22].

Herein we report clinical manifestations, immune features, genetic findings and type of treatment from the first Italian cohort of APDS-1 patients.

## 2. Materials and Methods

### 2.1. Patients

We describe eight patients (identified from P1 to P8) with a diagnosis of APDS-1 regularly followed at the following Italian Immunology Units: Pediatrics Unit, Monsignor Dimiccoli Hospital, Barletta; Pediatrics Clinic, ASST Spedali Civili of Brescia, University of Brescia, Brescia; Pediatric Hematology Department, MBBM Foundation, University of Milano Bicocca, Monza; Pediatric Oncology and Hematology, University of Pisa, Pisa; Pediatric Oncohematology Unit, SS Annunziata, Taranto. Six out of eight patients are of Italian origin, while another two are of eastern European origin (Romanian and Kosovar descendent, P5 and P7 respectively). All patients live in Italy. Retrospective data regarding medical history, clinical and laboratory data were obtained from analysis of medical notes. P2, P4, P5, P8 have already been described as single case reports [23,24,25,26], follow-up was therefore extended up to the last clinical visit. All patients (or their caregivers in case of pediatric patients) signed an informed consent form for this study according to the Hospital local ethical committee recommendations. The study was approved by the Hospital ethic committee (NP2972).

### 2.2. Genetic and Flow Cytometry Analysis

APDS-1 diagnosis was based on genetic analysis of *PIK3CD* gene. Genomic DNA was extracted from whole blood and the genetic analysis was performed using standard techniques. In previously undescribed variants, functional assays evaluating phospho-S6 Kinase (pS6K) levels were performed; briefly, peripheral CD4 and CD8 T cells of patients and healthy controls were stimulated with antiCD3 antibody for 24 h and then pS6K expression level was evaluated using anti-phosphoS6 235–236 antibody (Cell Signaling), and then analyzed by flow cytometry. In addition, CAL-101 inhibitor was used to restore the physiological level of pS6K expression. Data were analyzed by GraphPad Prism Software (version 8.0.0, GraphPad Software, Inc., San Diego, CA, USA) and statistical analysis was performed using the Student’s *t*-test. Patients with increased levels of pS6K were considered as carriers of a GOF mutation and therefore diagnosed as APDS-1. Whole blood was stained for immunophenotypic analysis using standard multiparametric flow cytometry protocols. Lymphocyte subset analyses were performed with a combination of monoclonal antibodies (Becton Dickinson, San Diego, CA, USA) according to the manufacturer’s instructions and completed by using the FlowJo software version 8.8.7 (TreeStar, Ashland, OR, USA). Immunological parameters were compared with the reference values based on a database obtained from a pool of age-matched healthy subjects.

## 3. Results

### 3.1. Patients

We report on eight patients with a molecular diagnosis of APDS-1, five males and three females. All the patients are alive. Tables 1 and 2 summarize demographic and clinical data of the patients.

The age at clinical onset was variable (0.5 to 20.5 years, median 3.95 years), with the vast majority of patients presenting symptoms since early in childhood. Molecular diagnosis of APDS-1 was achieved at a variable age (2.2 to 43.2 years, median 12.9 years). Diagnostic delay was considered as time span between first immunological evaluation and molecular diagnosis of APDS-1, ranging from 0.1 to 16.2 years (median 0.8 years). Median follow-up time was 3.5 years (1.0 to 21.7 years).

For four (P1, P5, P6, P7) out of eight patients lymphoproliferation (i.e., unexplained persistent or recurrent lymphadenopathies eventually associated with hepato- and/or splenomegaly) was the symptom leading to immunological evaluation, while two of them (P2, P3) further developed polyclonal lymphoproliferation during follow-up.

### 3.2. Infections in APDS-1 Patients

Sinopulmonary infections were the most common infectious complications in our cohort. Seven (P1, P2, P3, P4, P6, P7, P8) out of eight patients suffered from recurrent infections of both upper and lower respiratory tract, such as otitis media and pneumonia, since early in childhood. For four patients (P2, P3, P4, P8) a clinical history of recurrent respiratory tract infection (RRTI) was the symptom leading to the first immunological evaluation. Despite the high frequency of recurrent pneumonias in our population, only one patient (P2) developed pulmonary complications (i.e., bronchiectasis); the remaining patients (P1, P3, P4, P6, P7, P8), routinely examined with chest computed tomography (CT) scan and functional lung tests, remained complications-free. Four patients experienced severe infectious episodes requiring hospitalization for intravenous therapy and medical support: P1, bacterial sepsis, pathogen isolated: *H. influenzae*; P2, recurrent bacterial pneumonia with bilateral pleural effusion (no pathogen isolated); P7, left lobar pneumonia (no pathogen isolated); P8, recurrent otitis, pathogen isolated: *H. influenza, M. catarrhalis, P. aeruginosa*. At the time of the lobar pneumonia, P7 was on Sirolimus, which was discontinued during the hospitalization due to the increased infectious susceptibility.

*Epstein-Barr virus (EBV)* and *Cytomegalovirus (CMV)* viral loads were monitored at each clinical visit of the patients (from every 4–6 months to once a year, according to clinical needs). From the age of 17 months, P8 presented with chronic low-level EBV viremia (total viral load ranging from negative to 506 copies/mL; EBV viral-capsid antigen (VCA) IgM ranging from 43 AU/mL to 186 AU/mL). None of the other patients suffered from severe or recurrent viral infections, even though four (P1, P2, P3, P6) out of eight patients experienced occasional viral infections (respectively: *Varicella Zoster Virus, EBV, Herpes Simplex Virus 1, CMV*), all four patients were confirmed to be free of virus on subsequent Polymerase Chain Reaction testing. Only P1 and P3 required oral antiviral treatment in order to clear the infection.

Except for one patient (P1), who reported diverse episodes of oral candidiasis responsive to topical antimycotic treatment (probably related to inhaled steroid treatment for asthma control), we did not observe any other recurrent or invasive fungal infections in our population.

### 3.3. Lymphoproliferation in APDS-1 Patients

**Figure 1:**
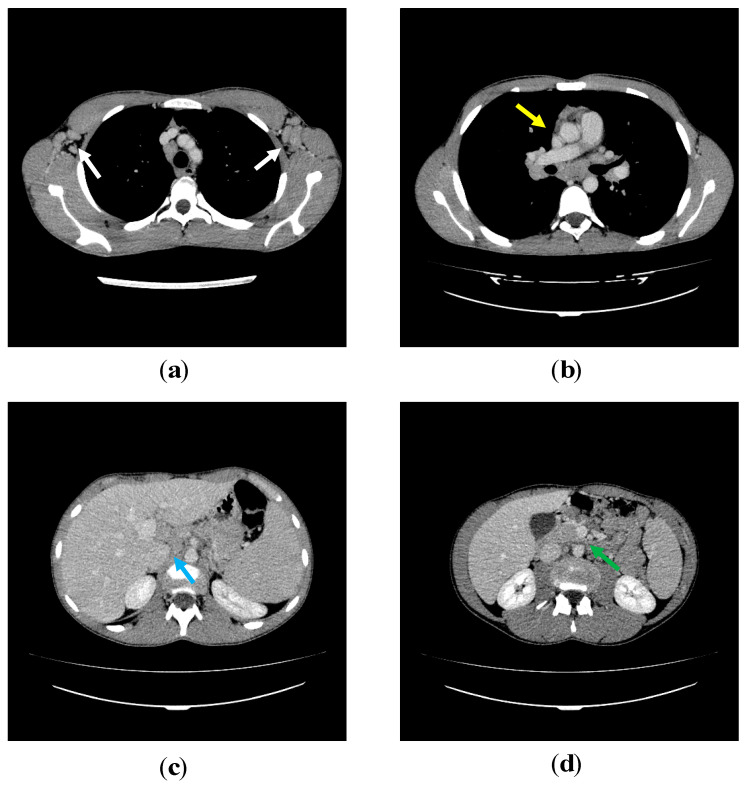
P2′s thoraco-abdominal computed tomography (CT) scans. On post contrast axial CT scans at the level of the axillary regions (**a**) numerous lymph nodes (white arrows) with homogeneous density are evident. On the scan just below the tracheal carina (**b**); solid mediastinal adenopathic conglomerate and bilateral hilar lymph nodes (yellow arrow) are clearly visible. In the abdomen, multiple adenopathies are evident in the hepatic hilum (**c**) (blue arrow) and in the mesentery (**d**) (green arrow).

**Figure 2:**
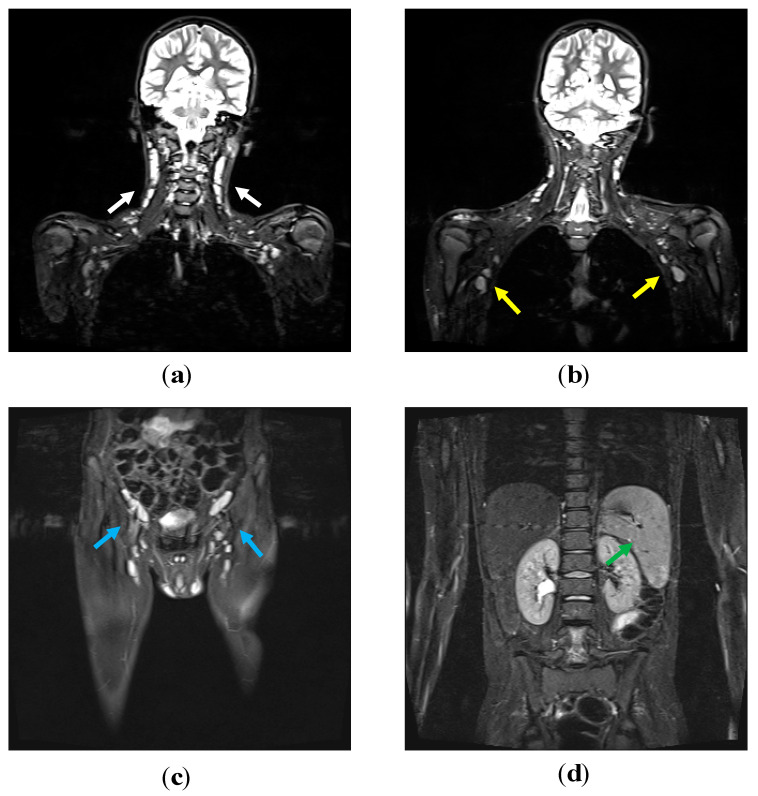
P5′s whole-body magnetic resonance imaging (MRI). On coronal fat suppressed T2 weighted sequences, numerous homogeneously hyperintense lymph nodes are detectable at the level of the neck (**a**) (white arrows), the axillary regions (**b**) (yellow arrows) and in the inguinal area bilaterally (**c**) (blue arrows). The spleen has a homogeneous signal intensity and increased size (**d**) (green arrow).

Lymph nodes, liver and spleen size were measured in our patients by thoracic and abdominal Computed Tomography and/or Magnetic Resonance Imaging at diagnosis and during follow-up (every five years or more frequently based on clinical needs) (Figure 1; Figure 2, thoraco-abdominal CT scan of P2 and whole-body MRI of P5). Seven out of eight patients exhibited chronic benign lymphoproliferation: six of them (P1, P2, P3, P4, P5, P6, P7) had diffuse lymphadenopathies and hepatosplenomegaly and one (P8) had chronic cervical lymphadenopathies and splenomegaly.

For four patients (P1, P5, P6, P7), lymphoproliferation was the symptom that led to immunological evaluation. During follow-up, P2 and P3 began to develop signs and symptoms suggestive of lymphoproliferative disease. In one case (P5), lymphoproliferation was complicated by hemophagocytic lymphohistiocytosis (HLH). Functional and genetic analysis ruled out familiar HLH and other possible triggers were excluded (i.e., malignancy, infections) [25]. All our patients underwent an extended diagnostic work-up in order to exclude malignancy. Immunohistochemical analysis of bioptic specimens of all the six patients with diffuse lymphadenopathies were suggestive of reactive, non-neoplastic, polyclonal benign lymphoproliferation (Table 3).

No lymphomas nor any other malignancies have been reported in our population up to date.

### 3.4. Other Clinical Features

Four patients (P2, P3, P5, P7) presented with autoimmune or autoinflammatory manifestations. At 12 years of age, P2 developed psoriatic dermatitis. P3 onset was characterized by erythema nodosum, and during the follow-up period developed subclinical autoimmune thyroiditis, leuco-thrombocytopenia, and Chron’s-like inflammatory bowel disease. P5 presented at onset with clinical history of migrant arthritis and rash further complicated by secondary HLH. During follow-up, P7 suffered from recurrent parotiditis and experienced one episode of hemolytic anemia. Routinely checked for autoimmune manifestation, none of the other patients developed autoantibodies or other autoimmune features.

Three out of eight patients (P1, P3, P6) developed asthma during follow-up, easily controlled with inhaled steroid therapy. During her first years of life, in occurrence with respiratory tract infection, P8 experienced concomitant wheezing episodes.

In strong contrast with the rest of the cohort, P4 had an atypically mild clinical course: besides the progressive development of B cell lymphopenia, she only suffered from occasional respiratory infections responsive to oral antibiotics, and remained persistently negative for EBV and CMV, without any signs of lymphoproliferation [24].

At the time of her first immunological evaluation, P7 presented with growth and puberty delay.

None of our patients showed and/or developed neurodevelopmental impairment.

### 3.5. Immunological Features at Onset

Immunological data of the patients at the time of their first evaluation are depicted in Table 4. Decreased serum IgG levels were detected in three patients (P3, P4, P6) at the time of diagnosis. Two patients (P1, P6) presented with increased serum IgM levels; serum IgA levels were reduced in three (P4, P6, P8) out of eight patients. We evaluated specific antibody response to tetanus and Hepatitis B Surface antigen (HbsAg) vaccination: four patients showed undetectable anti-Tetanus IgG and anti-HbsAg IgG, while the other patients’ titer was found to be protective. In addition, P2 presented a selective antibody defect to polysaccharide antigens [23].

Lymphocyte immunophenotyping findings at onset are pictured in Table S1 (Available Online at www.mdpi.com/xxx/s1). Common findings on T-lymphocyte subsets were a low CD4^+^/CD8^+^ ratio with reduced CD4^+^ T-cell counts and CD8^+^ cells skewed towards effector/memory (CD8^+^CD45RA^–^CCR7^–^). CD4^+^ cells reduction was particularly affected on naïve cells (CD4^+^CD45RA^+^CCR7^+^) and Recent Thymic Emigrant cells (RTE) (CD45RA^+^CCR7^+^ CD31^+^). When tested, we confirmed a raised percentage of CD57^+^ and CD8^+^CD57^+^ cells, a cluster of differentiation for senescent cells. T cells presented an increased activation state, with raised HLA-DR^+^ both on CD4^+^ and CD8^+^ T-cells. We also observed an impairment on different steps in B cells development and maturation, with markedly increased CD19^hi^CD21^low^.

T cells proliferation upon stimulation with anti-CD3, anti-CD3+IL-2 and Phytohemagglutinin (PHA) was evaluated in three patients. P1 showed a markedly reduced proliferation upon stimulation with PHA and P6 showed a slightly reduced proliferation index upon stimulation with anti-CD3 and PHA only in CD8^+^ cells, while P3 test was normal, compared to healthy donor. The remaining five patients (P2, P4, P5, P7, P8) were on steroid treatment and thus T cells proliferation was not evaluated.

### 3.6. PIK3CD Genetic Analysis and Phospo-S6 Kinase Assays

*PIK3CD* genetic analysis revealed five distinct mutations (Table 5). Mutations detected in P1, P4 and P5 are novel missense.

**Figure 3:**
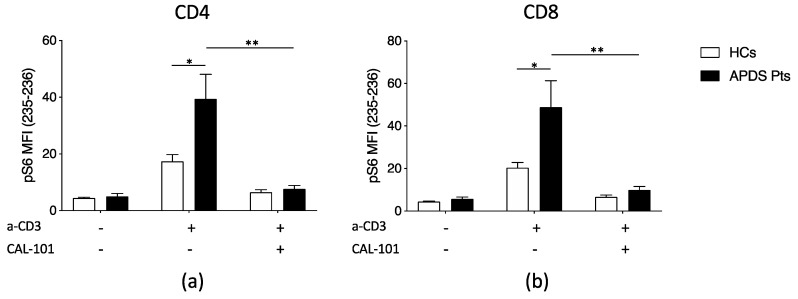
phospo-S6 kinase (pS6K) levels in peripheral T cells from Activated Phosphoinositide 3-Kinase Delta Syndrome-1 (APDS-1) patients (Pts). Summarized data for pS6K levels from patients harbouring the Y524D, P658L and R108L PIK3CD mutations are shown for CD4^+^ T cells (**a**) and CD8^+^ T cells (**b**) after anti-CD3 stimulation and/or CAL-101 treatment. (Data were summarized from *n* = 3 experiments from the index patients and four different healthy controls (HCs); statistical analysis was performed using the Student’s t-test (* *p* < 0.05). Abbreviations: a-CD3: anti-CD3, CAL-101: Idelalisib, GS-1101, MFI: mean fluorescence intensity.

In P1 we detected a missense mutation in the helical domain (c.1570T > G) that resulted in a Tyrosine to Aspartic Acid substitution (*p*.Y524D). In P4, we identified and already described a missense mutation (c.1973C > T) in the highly conserved P658 region resulting in a Proline to Leucine variation (*p*.P658L) [24]. In P5, a novel missense mutation in c.323C > G (*p*.R108L) was found, involving the linker region of the adaptor binding domain [25]. We confirmed the activating nature of the three variants detected in P1, P4 and P5 comparing the phosphorylation pattern of S6K of the patients with healthy controls and patients harboring the classical E1021K mutation: patients’ T-cells showed a significantly increased phosphorylation of S6K when stimulated with a-CD3, which could be downregulated by treating T-cells with CAL-101 (Idelalisib, GS-1101), a selective p110δ in vitro inhibitor Figure 3). In the remaining five patients, four (P2, P3, P6, P8) presented the previously described hotspot mutation c.3061G > A (*p*.E1021K), and one (P7) the already described c.1574A > C (*p*.E525A).

### 3.7. Therapeutic Strategies

Three patients (P3, P4, P6) presented with hypogammaglobulinemia, undetectable titer to the tested vaccines (i.e., Tetanus, Hepatitis B Vaccine) and impaired B-cells maturation, thus were started on immunoglobulin replacement treatment (IgRT). Although with normal IgG levels, P1, P2, P8 were put on IgRT based on their clinical manifestation (P1—recurrent pneumonia and a bacterial sepsis; P2—recurrent pneumonia with bronchiectasis; P8—recurrent pneumonia).

Systemic antibiotic and antiviral treatments were used in case of infectious episodes. One patient (P3) was put on Co-Trimoxazole prophylaxis. None of the other patients required either antibiotic or antiviral or antifungal prophylaxis.

Oral steroid cycles were often used in four patients (P1, P2, P5, P6) in order to control lymphoproliferative symptoms. Secondary HLH was treated with high dose intravenous steroid therapy [25].

When P3 developed enteropathy, he was initially treated with mesalazine, oral steroids, and probiotics cycles; due to lack of clinical response to first line treatment, anti-TNF-α (Infliximab) was undertaken.

In two patients (P7, P8) sirolimus was used to control lymphoproliferation, with clinical and radiological improvement. P7 reported an increased rate of RRTI and thus the drug was stopped.

In P7, and despite initial response to medical treatment, continuous and severe infectious episodes compromised her quality of life, and thus led us to propose HSCT: at 14.0 years, P7 underwent HSCT from a 10/10 Human Leukocyte Antigen Matched Unrelated Donor. The patient received a myeloablative regimen with treosulphan, fludarabine and anti-thymocyte globulin; Graft-versus-Host Disease (GvHD) prophylaxis was based on cyclosporin A and methotrexate; Rituximab was used as lymphoproliferation prophylaxis. HSCT was not complicated by GvHD nor infectious episodes. At 21 months post-HSCT, she presents mixed chimerism (94%), stable hematological values with resolution of infections and lymphoproliferation.

## 4. Discussion

We present clinical and immunological data of the Italian cohort of APDS-1 patients with long term follow-up.

As with other inborn errors of immunity (IEI), APDS-1 may manifest during both pediatric and adult age: half of our population was diagnosed in childhood, with an apparently positive impact in terms of quality of life and complications management. Six out of eight patients presented symptoms before age 8, in particular infectious episodes.

Based on the symptoms that leaded to first immunological evaluation, an initial diagnostic hypothesis of IEI was posed in five out of eight patients (combined immunodeficiency, CVID/CVID-like or HLH), while the remaining three patients were initially considered has having lymphoma.

Infectious episodes, in particular affecting the upper and lower respiratory tract, occurred in the majority of our cohort (7/8 patients), since early in childhood. While persistent or recurrent *Herpesviridae* infections represented a key feature of previously described APDS-1 patients [8], in our cohort only one patient had persistent EBV viremia. In the remaining four patients with an occasional viral infectious episode, only P1 and P3 required a course of oral antiviral treatment in order to clear the infection.

Chronic benign lymphoproliferation is considered a hallmark of APDS-1. In our cohort, all patients except one experienced recurrent episodes of lymphoproliferation. The six patients (P1, P2, P3, P5, P6, P7) with diffuse lymphadenopathies underwent a complete diagnostic work-up in order to exclude malignancy: histological findings were consistent with reactive non-infectious benign lymphoid hyperplasia. Except for P6′s lymph node biopsy, which tested EBV positive, we did not record any particular trigger (e.g., viral infection) for the remaining patients. Even though it is widely assumed that patients with primary immune deficiencies have an increased risk in developing hematological malignancies [28], we did not record any malignancies in our cohort. This could be partially explained by the paucity of the population or the relatively short length of the follow-up.

Apart from infections and lymphadenopathies, some of our patients presented with unusual manifestations that extend our knowledge on APDS-1 onset and natural history. We report for the first time a patient with HLH secondary to APDS-1-related lymphoproliferation. Functional and genetic analysis for familiar HLH tested negative and other HLH-related triggers were excluded (i.e., malignancy, infections). Another APDS-1 patients has been described with Hodgkin Lymphoma complicated by HLH [29], underlining a pivotal role of the PI3K axis in terms of oncogenesis and immune regulation. In addition, Chron’s-like enteropathy, a feature shared by many other IEI (i.e., Cytotoxic T-Lymphocyte Antigen 4 (*CTLA-4*) haploinsufficiency, immunodysregulation polyendocrinopathy enteropathy X-linked (IPEX), Lipopolysaccharide-Responsive, Beige-Like Anchor Protein *(LRBA)* deficiency) [30,31], was reported in one of our patients and initially responded to first-line treatment [32]. Data regarding second-line treatment in APDS-1-related enteropathy are lacking and a better therapeutical approach in this lesser frequent complication needs to be identified.

Reduced serum IgG levels were present in three patients (P3, P4, P6): they were put on IgRT with positive reduction of the infectious episodes. Another three patients (P1, P2, P8), even though not presenting with hypogammaglobulinemia, due to the occurrence of severe infectious episodes were put on IgRT. In addition, P2 presented a selective antibody defect to polysaccharide antigens and P8 partial IgA deficiency. These findings suggest that IgRT in APDS-1 patients should be carefully evaluated, based not only on immunological findings but also on clinical history.

As already known, the hyperactivated mTOR pathway in APDS-1 leads to an increased T-cell exhaustion, in particular CD8^+^ T cells [16]. When evaluated, we confirmed increased T-cell senescence, characterized by the expression of CD57^+^. In addition, CD4^+^ cells appear to be reduced, with a decrease in thymic output (naïve cells and RTE) and T cells skewed towards activated subsets. Both CD4^+^ and CD8^+^ T-cells showed also an increased percentage of activated cells expressing HLA-DR^+^. B-cells alterations appear to be more variable, with different degrees of maturation impairment. In particular, we observed that the vast majority of patients presented elevated CD19^hi^CD21^low^, a subset known to be associated with autoimmunity or immune dysregulation [33]. Moreover, 5/8 patients (P2, P4, P5, P6, P8) presented low to absent switched memory B cells (IgD^−^IgM^−^CD27^+^). While T cell alterations are constant and recurrent in APDS-1 patients, perturbations in B cell phenotype and Ig serum levels do not appear equally constant and recurrent, suggesting that *PIK3CD* gene analysis may be undertaken in patients with recurrent infections, lymphoproliferation, and aberrant T-lymphocyte distribution with increased senescence and activation, even in the absence of hypogammaglobulinemia or CVID-like phenotype. In fact, and while the latest International Union of Immunological Societies Expert Committee Classification of Human Inborn Errors of Immunity Update included APDS-1 in IEI characterized by predominantly antibody deficiency [34], our cohort’s phenotype overlaps more with other IEI belonging to the group of immune dysregulation, such as the aforementioned *CTLA-4* haploinsufficiency, *LRBA* deficiency or the autoimmune lymphoproliferative syndrome group. Thus, it becomes evident that APDS-1 should be considered in the diagnostic work-up of various immunological conditions, even in the absence of hypogammaglobulinemia.

Our APDS-1 cohort presents a wide genetic heterogeneity. Functional analysis confirmed the GOF effects of these variants. While the *p*.E1021K remains one of the most frequent disease-related variant, novel variants in different regions of the *PIK3CD* gene, as the ones reported here, may offer useful insights on the biological role of different *PIK3CD* genetic regions in the PI3K hyper-activation cascade.

IgRT has proven to be a mainstay in patients with predominantly antibody defects, not only for infection prevention but also for its immunomodulating activity [35]. As mentioned before, IgRT should be considered in any APDS-1 patients with clinical history of severe or recurrent infectious episodes, even if not hypogammaglobulinemic. Oral steroid therapy has proven short term efficacy in controlling lymphadenopathies, while on the long-term is known to have more side effects than benefits [36]. Sirolimus showed great efficacy in controlling lymphoproliferation, as already reported by the European Society for Immunodeficiencies APDS registry [9]. Although it increases the infective risk, the use of mTOR inhibitors should be strongly considered as a steroid sparing agent. The introduction of selective p110δ inhibitors is opening possible new scenarios for the treatment of affected patients [20]. HSCT has been used in a limited number of cases, although with an elevated risk of complications [21,22].

In conclusion, our data underline that APDS-1 has wide variability in clinical and immunological features, ranging from barely asymptomatic patients to more severe and life-threatening forms. Novel mutations have been described, with functional confirmation of their causative role offering additional insights in B- and T-cell physiology. Besides immunoglobulin replacement treatment, HSCT may represent a life-saving option for a limited number of cases while therapies targeting the PI3K pathway will most likely become a valid aid for the amelioration of patients’ clinical management and their quality of life.

## Figures and Tables

**Table 1:** Demographical data of eight Italian APDS-1 patients.

	P1	P2	P3	P4	P5	P6	P7	P8
Gender	Male	Male	Male	Female	Male	Male	Female	Female
Status at last clinical visit	Alive	Alive	Alive	Alive	Alive	Alive	Alive	Alive
Current age *	20.7	29.7	14.0	48.8	12.8	9.3	15.8	6.5
Follow-up time ^†^	1.0	16.7	7.0	21.7	1.0	4.0	3.0	2.5
Onset of infection *	4.5	0.5	0.7	20.5	–	3.4	8.0	1.0
Onset of lymphoproliferation *	19.0	27.7	11.0	–	12.0	5.2	8.0	1.5
Onset of autoimmunity *	–	12.0	4.7	–	11.2	–	13.0	–
First immunological evaluation ^§^	2019	2002	2011	1998	2019	2016	2017	2016
First immunological evaluation *	19.7	12.0	4.7	27.0	12.0	5.3	13.2	1.7
APDS-1 diagnosis *	19.7	29.0	7.6	43.2	12.0	5.3	13.8	2.2
Diagnostic delay ^†^	0.7	15.0	2.9	16.2	0.8	0.1	0.8	0.7

Abbreviations: APDS-1: Activated Phosphoinositide 3-Kinase Delta Syndrome-1. Notes: * Age in years; †: time span in years; §: year.

**Table 2:** Clinical data of eight Italian APDS-1 patients.

	P1	P2	P3	P4	P5	P6	P7	P8
Symptom leading to immunological evaluation	Lymphoproliferation	RRTI	EN, Pneumonia	RRTI	HLH	Lymphoproliferation	Lymphoproliferation	RRTI
Initial diagnostic hypothesis	Lymphoma	CVID-like	CVID	CVID	HLH	Lymphoma	Lymphoma	CID
Sinopulmonary infections	Otitis, Pneumonia,	Pneumonia	Pneumonia	Otitis, Pneumonia	–	Otitis, Pneumonia	Pneumonia	Otitis, Pneumonia
Infections other than URTI	Sepsis, episodic Candidiasis, recurrent	Dental abscess Gastroenteritis	–	–	–	–	Otomastoiditis	–
Viral infection	VZV, episodic	EBV, episodic	HSV-1, episodic	–	–	CMV, episodic	–	EBV, persistent
Lymphadenopathies	Diffuse	Diffuse	Diffuse	–	Diffuse	Diffuse	Diffuse	Cervical
Hepato/splenomegaly	Hepatosplenomegaly	Hepatosplenomegaly	Hepatosplenomegaly	–	Hepatosplenomegaly	Hepatosplenomegaly	Hepatosplenomegaly	Splenomegaly
Autoimmunity	–	Psoriatic dermatitis	EN, Leucopenia, Thrombocytopenia, Thyroiditis	–	Arthritis, Rash	–	Hemolytic anemia Recurrent parotiditis	–
Allergy and asthma	Asthma	–	Asthma	–	–	Asthma	–	Wheezing
Gastrointestinal involvement	–	–	IBD	–	–	–	–	–
Neurodevelopmental delay	–	–	–	–	–	–	–	–
Others	–	–	–	–	–	–	FTT, Delayed puberty	–
Malignancy	–	–	–	–	–	–	–	–

Abbreviations: APDS-1: Activated Phosphoinositide 3-Kinase Delta Syndrome-1, CID: combined immune deficiency, CMV: cytomegalovirus, CVID: common variable immune deficiency, EBV: Epstein–Barr virus, EN: erythema nodosum, FTT: failure to thrive, HLH: hemophagocytic lymphohistiocytosis, HSV-1: herpes simplex virus 1, IBD: inflammatory bowel disease, RRTI: recurrent respiratory tract infection, VZV: varicella zoster virus.

**Table 3:** Histological analysis of lymph nodes of eight Italian APDS-1 patients.

	P1	P2	P3	P4	P5	P6	P7	P8
Date of biopsy	2019	2019	2017	/	2019	2016	2017	/
Lymph node region	Cervical	Cervical	Cervical	/	Inguinal	Cervical	Axillary	/
Lymph node histology	Atypical polyclonal lymphoproliferation, EBV-negative	Polyclonal paracortical hyperplasia, EBV-negative	Reactive lymphoproliferation	/	Reactive predominantly paracortical hyperplasia, HSV-1, HSV-2, CMV, EBV negative	Atypical marginal zone hyperplasia with scattered EBV^+^ cells	Interfollicular hyperplasia	/

Abbreviations: APDS-1: Activated Phosphoinositide 3-Kinase Delta Syndrome-1, CMV: cytomegalovirus, EBV: Epstein–Barr virus, HSV-1: herpes simplex virus-1, HSV-2: herpes simplex virus-2.

**Table 4:** Immunological data of eight Italian APDS-1 patients at first evaluation.

		P1	P2	P3	P4	P5	P6	P7	P8
WBC ×10^3^/µL (4.0–10.8)		11.25	6.90	7.18	4.18	6.14	8.8	8.51	8.06
Neutrophils ×10^3^/µL (1.5–8.0)		9.40	3.40	4.93	2.93	4.58	5.33	6.43	4.90
Lymphocytes ×10^3^/µL (0.9–4.0)		1.51	2.30	1.42	0.785	1.21	2.38	1.27	2.12
IgG, mg/dL (700–1600)		855	1452	282 ^†^	495	2119 ^ß‡^	362 ^ƒ§^	2056 ^ß‡^	725 ^∂#^
IgA, mg/dL (70–400)		227	80	55 ^†^	10	207 ^ß‡^	38 ^ƒ§^	287 ^ß‡^	15 ^∂#^
IgM, mg/dL (40–230)		2332	145	170 ^†^	7	127 ^ß‡^	414 ^ƒ§^	155 ^ß‡^	228 ^∂#^
Anti-HBs IgG (>10 IU/L)		30 IU/L	*n*.a.	absent	absent	584 IU/L	absent	*n*.a.	*n*.a.
Anti-tetanus IgG (>0.1 IU/mL)		0.5 IU/L	2.5 IU/L	absent	absent	0.6 IU/mL	absent	*n*.a.	1.0 UI/L
CFSE-base T cells Proliferation assay									
	Anti-CD3	normal	*n*.a.	normal	*n*.a.	*n*.a.	CD4^+^ normalCD8^+^ reduced	*n*.a.	*n*.a.
	Anti-CD3 + IL-2	normal	*n*.a.	normal	*n*.a.	*n*.a.	normal	*n*.a.	*n*.a.
	PHA	reduced	*n*.a.	normal	*n*.a.	*n*.a.	CD4^+^ normalCD8^+^ reduced	*n*.a.	*n*.a.

Abbreviations: Anti-HBs IgG: Anti-Hepatitis B surface Immunoglobulin G, APDS-1: Activated Phosphoinositide 3-Kinase Delta Syndrome-1, CFSE: Carboxyfluorescein succinimidyl ester, IL-2: Interleukin-2, IgA: immunoglobulin A, IgG: immunoglobulin G, IgM: immunoglobulin M, PHA: Phytohemagglutinin, WBC: White Blood Cells. Notes: *n*.a.: not available; †: age adjusted reference value: IgG 528–1959 mg/dL, IgA 37–257 mg/dL, IgM 49–292 mg/dL; ß‡: age adjusted reference value: IgG 640–1909 mg/dL IgA 61–301 mg/dL, IgM 59–297 mg/dL; ƒ§: age adjusted reference value: IgG 633–2016 mg/dL, IgA 41–315 mg/dL, IgM 56–261 mg/dL; ∂#: age adjusted reference value: IgG 462–1710 mg/dL, IgA 27–173 mg/dL, IgM 62–257 mg/dL.

**Table 5:** Genetic analysis and phospo-S6 Kinase evaluation of eight APDS-1 patients.

Patients No.	PIK3CD_MUT	PIK3CD_EFF	pS6K
1	c.1570T > G	*p*.Y524D	Increased
2	c.3061G > A	*p*.E1021K	Increased *
3	c.3061G > A	*p*.E1021K	Increased *
4	c.1973C > T	*p*.P658L	Increased *
5	c.323C > G	*p*.R108L	Increased *
6	c.3061G > A	*p*.E1021K	Increased *
7	c.1574A > C	*p*.E525A	Increased *
8	c.3061G > A	*p*.E1021K	Increased *

Abbreviations: APDS-1: Activated Phosphoinositide 3-Kinase Delta Syndrome-1, PIK3CD_EFF: PIK3CD effect, PIK3CD_MUT: PIK3CD mutation, pS6K: phospo-S6 Kinase. Note: * As previously reported [1,2,24,25,27].

## Supplementary Materials

The following are available online at https://www.mdpi.com/2077-0383/9/10/3335/s1: Table S1: Lymphocyte immunophenotyping of eight APDS-1 patients.
