# Supplementary material for: Activated Phosphoinositide 3-Kinase Delta Syndrome 1: Clinical and Immunological Data from an Italian Cohort of Patients
Source: J Clin Med. 2020 Oct 17;9(10):3335. doi: 10.3390/jcm9103335 (PMC7603210; doi:10.3390/jcm9103335)
Supplement: Supplementary file 1 [file jcm-09-03335-s001.pdf]

Journal of Clinical Medicine

MDPI

Supplementary materials

Table 1. Lymphocyte immunophenotyping of eight APDS-1 patients.

	P1		P2		P3		P4		P5		P6		P7		P8	
	Linf%	Par%	Linf%	Par%	Linf%	Par%	Linf%	Par%	Linf%	Par%	Linf%	Par%	Linf%	Par%	Linf%	Par%
T Lymphocytes (CD3$^+$)	87.7↑		49.6↓		78.0		88.7↑		86.8↑		81.4↑		34.9↓		72.2	
CD3$^+$CD4$^+$	21.8↓		17.9↓		30.0		59.2		25.1↓		21.5↓		15.2↓		22.9↓	
CD4$^+$HLA-DR$^+$		12.4↑		25.7↑		n.a.		4.9		7.1		14.9↑		n.a.		15.2
Naïve (CD45RA$^+$CCR7$^+$)		12.4↓		8.1↓		29.0↑		22.1		43.9		8.2↓		10.0↓		24.8↓
RTE (CD45RA$^+$CCR7$^+$CD31$^+$)		6.4↓		3.6↓		0.0↓		19.5		26.5		5.8↓		5.2↓		20.5↓
Centr. Mem. (CD45RA$^-$CCR7$^+$)		30.4		29.1		15.0		16.9		15.2		35.3		61.6↑		24.1
Eff. Mem. (CD45RA$^-$CCR7$^-$)		52.0↑		59.8↑		71.0↑		60.3↑		32.0↑		54.3↑		28.1↑		43.1↑
Term. Diff. (CD45RA$^+$CCR7$^-$)		5.2		3.0		0.0↓		0.6		8.8↑		2.2		0.3↓		8.3↑
CD3$^+$CD8$^+$	62.0↑		27.7		23.0		21.2		58.3↑		53.2↑		26.27		41.7↑	
CD8$^+$HLA-DR$^+$		18.5		47.3↑		n.a.		7.8		25.2↑		19.0		n.a.		16.7
Naïve (CD45RA$^+$CCR7$^+$)		1.3↓		3.4↓		15.0↓		30.3		17.7		2.3↓		9.8↓		2.4↓
Centr. Mem. (CD45RA$^-$CCR7$^+$)		0.2↓		0.5↓		52.0↑		3.5		0.2↓		0.2↓		37.8↑		4.0
Eff. Mem (CD45RA$^-$CCR7$^-$)		38.3		39.1		33.0		24.5		42.0↑		31.9		38.8↑		41.9↑
Term Diff. (CD45RA$^+$CCR7$^-$)		60.2		57.0		0.0↓		41.6		40.1		65.5↑		13.7		52.3
CD4$^+$/CD8$^+$	0.35↓		0.65↓		1.3		0.3↓			0.43↓	0.4↓		0.6↓		0.3↓	
TCR Gamma/Delta	2.8		2.8		19.0↑		4.6		3.1		10.6		n.a.		n.a.	
CD57$^+$	44.8↑		33.4↑		16.0↑		n.a.		25.3↑		n.a.		n.a.		n.a.	
CD8$^+$CD57$^+$		35.5↑		10.6↑		11.0↑		n.a.		16.2↑		n.a.		n.a.		n.a.
B Lymphocytes (CD19$^+$)	7.4		8.7		4.0		0.4↓		1.5↓		12.5		0.9↓		8.9↓	
RBE (CD38^{hi}CD10$^+$)		4.3		46.0↑		0.0↓		21.8		40.5↑		57.7↑		n.a.		41.8
Naïve (IgD$^+$CD21^{hi}CD10$^-$CD27$^-$)		32.9↓		5.0↓		0.0↓		56.9		0.0↓		6.1↓		36.4↓		10.2↓
CD19^{hi}CD21^{low}		31.9↑		21.0↑		62.0↑		20.4↑		6.7		10.6↑		13.7↑		13.2↑
Switched Mem. (IgD$^-$IgM$^-$CD27$^+$)		13.3		0.8↓		6.0		0.0↓		0.0↓		0.3↓		30.3↑		0.0↓
IgM Mem. (IgD$^+$IgM$^+$CD27$^+$)		8.7		2.4↓		19.0↑		0.9↓		0.0↓		0.4↓		n.a.		4.1↓
Term. Diff. (CD38^{hi}CD27^{hi}CD20$^-$)		6.3		20.8↑		0.0↓		0.0↓		45.9↑		22.3↑		n.a.		27.8↑
NK Cells																
NK Cells (CD3$^-$CD16$^+$CD56$^+$)	4.6		40.7↑		5.0		8.6		11.5		5.7		35.4↑		15.7	

Abbreviations: APDS-1: Activated Phosphoinositide 3-Kinase Delta Syndrome-1, Centr. Mem.: Central Memory, Eff. Mem.: Effector Memory, IgM: immunoglobulin M, NK: Natural Killer, RBE: Recent Bone Marrow Emigrants, RTE: Recent Thymic Emigrants, Switched Mem.: Switched Memory, TCR: T-cell receptor, Term. Diff.: Terminally Differentiated. Notes: n.a.: not available; ↓: lower than a pool of age-specific healthy subjects; ↑: higher than a pool of age-specific healthy subjects.
